# CDKN1C as a prognostic biomarker correlated with immune infiltrates and therapeutic responses in breast cancer patients

**DOI:** 10.1111/jcmm.16880

**Published:** 2021-08-31

**Authors:** Jianguo Lai, Xiaoyi Lin, Fangrong Cao, Hsiaopei Mok, Bo Chen, Ning Liao

**Affiliations:** ^1^ Department of Breast Cancer Guangdong Provincial People’s Hospital,Guangdong Academy of Medical Sciences Guangzhou China

**Keywords:** breast cancer, CDKN1C, overall survival, prognosis, therapeutic response

## Abstract

Breast cancer (BC) prognosis and therapeutic sensitivity could not be predicted efficiently. Previous evidence have shown the vital roles of CDKN1C in BC. Therefore, we aimed to construct a CDKN1C‐based model to accurately predicting overall survival (OS) and treatment responses in BC patients. In this study, 995 BC patients from The Cancer Genome Atlas database were selected. Kaplan‐Meier curve, Gene set enrichment and immune infiltrates analyses were executed. We developed a novel CDKN1C‐based nomogram to predict the OS, verified by the time‐dependent receiver operating characteristic curve, calibration curve and decision curve. Therapeutic response prediction was followed based on the low‐ and high‐nomogram score groups. Our results indicated that low‐CDKN1C expression was associated with shorter OS and lower proportion of naïve B cells, CD8 T cells, activated NK cells. The predictive accuracy of the nomogram for 5‐year OS was superior to the tumour‐node‐metastasis stage (area under the curve: 0.746 vs. 0.634, *p* < 0.001). The nomogram exhibited excellent predictive performance, calibration ability and clinical utility. Moreover, low‐risk patients were identified with stronger sensitivity to therapeutic agents. This tool can improve BC prognosis and therapeutic responses prediction, thus guiding individualized treatment decisions.

## 1 INTRODUCTION

Following GLOBOCAN statistics 2020, breast cancer (BC) is the most prevalently diagnosed cancer and ranks first for mortality in women.^1^ The survival of BC survivors has been improved by early detection and rapid development of multimodal therapy, including locoregional and systemic management.^2^, ^3^ Nonetheless, some BC patients still face with undesirable survival outcomes owing to refractory therapeutic sensitivity and recurrence.^4^, ^5^, ^6^, ^7^ Characterized with biological heterogeneity, BC prognosis prediction that mainly relied on tumour‐node‐metastasis (TNM) staging and conventional molecular subtypes appear limitedly. In recent years, other prognostic indicators emerged to achieve higher accuracy of survival prediction. It is known that tumour‐infiltrating immune cells (TIICs) play a vital prognostic role in BC patients.^8^, ^9^, ^10^ For instance, high levels of tumour‐infiltrating lymphocytes generally indicated a favourable prognosis.^11^, ^12^ Another breakthrough is genetic sequencing widely applied in the individualized treatment and prognosis of BC patients.^13^, ^14^, ^15^ Genes are closely associated with cell cycle and apoptosis, thus playing pivotal roles in tumour progression. Numerous genome variants have been reported to be associated with BC survival outcomes and treatment responses.^16^, ^17^, ^18^ Antitumor medicine can decrease the risk of recurrence and BC mortality,^19^, ^20^ but its application was limited by the uncertain effectiveness and common adverse effects.^21^ Although a vast majority of methods had been generated to monitor the therapeutic responses,^22^, ^23^ they could not identify the patients who can benefit from some specific drugs clinically. Hence, the construction of a novel tool for precise prediction of BC prognosis and therapeutic responses is required.

CDKN1C, encoding the Cyclin‐dependent kinase inhibitor p57^Kip2^, is a paternally imprinted gene on chromosomal band 11 p15.5. Its encoded protein blocks the substrate interaction domain on cyclins and prevents binding of ATP and catalytic activity, thus mediating cyclin/CDK complex inhibition and negatively regulating cell proliferation.^24^ It can also cause cell cycle arrest via binding and inhibition of PCNA.^25^ As a tumour suppressor gene, CDKN1C is implicated in various human cancers and Beckwith‐Wiedemann Syndrome.^26^ Previous studies have tried to investigate the connection between CDKN1C and BC. Downregulation and hypermethylation of CDKN1C have been acknowledged prevalent in BC, which are related to a deterioration of prognosis.^27^, ^28^ With respect to therapeutic application, Y Ma et al. have revealed transcriptional upregulation of CDKN1C correlated with CDK inhibitors.^29^ Interestingly, some antioxidant agents and wellness interventions were also reported to increase the expression levels in BC cells.^30^, ^31^ In contrast, through epigenetic mechanisms, CDKN1C can be suppressed by methylation and histone deacetylation,^32^ multiple micro‐RNAs and lncRNAs,^33^, ^34^ and specifically ERα signalling in hormone‐responsive BC cells.^35^ These observations all support the implication of CDKN1C in BC tumorigenesis.

Despite the fact that BC harbouring lower levels of CDKN1C tended to present with poor survival outcomes,^36^ its role in BC progression and prognostic evaluation remained largely unknown. Therefore, we aimed to ascertain the CDKN1C expression and its relationship with prognosis in BC. Besides, the association between CDKN1C expression and enriched gene sets and pathways, as well as tumour immune microenvironment (TIM), were investigated in BC patients.

Recently, nomogram is widely conducted as a personalized tool to predict prognosis intuitively and precisely in various cancers.^37^, ^38^, ^39^, ^40^, ^41^, ^42^ Because this tool can rapidly calculate through easy‐to‐use digital interfaces and more easily acquire prognostic information compared with traditional TNM staging. Moreover, nomograms can integrate biological and clinicopathological parameters to establish a prognostic model that generates a possibility of survival outcome.

Thus, to improve the accuracy of survival and therapeutic sensitivity assessment for BC patients, a novel prediction model integrating the expression of CDKN1C was established. We aimed to build a CDKN1C‐based nomogram to predict overall survival (OS) and therapeutic responses in BC patients.

## 2 MATERIALS AND METHODS

### 2.1 Study samples from TCGA database

A total of 995 BC samples with specific CDKN1C expression levels were screened from the Cancer Genomes Atlas (TCGA) data portal. Patients without complete follow‐up data or whose survival period was shorter than 1 month were excluded. Other clinical and pathological characteristics included in our analysis were as follows: age at diagnosis, T, N and TNM stage, tumour subtype and survival time. In the light of the optimal cut‐off value of CDKN1C expression, study samples were classified into two groups with 786 patients in the low‐expression group and 209 patients in the high‐expression group.

### 2.2 Differential expression and survival analysis of CDKN1C

First, differential gene expression analysis of CDKN1C was performed based on TCGA database via a Sangerbox tool, including 1098 BC and 113 normal breast tissues. In order to assess the effects of differentially expressed CDKN1C on prognosis, Kaplan‐Meier survival analysis was utilized to estimate the OS of the TCGA patients. Subsequently, univariate and multivariate analyses were formulated to evaluate the prognostic effects of CDKN1C and other potential risk factors.

### 2.3 Gene set enrichment analysis (GSEA) and immune infiltrates analysis of CDKN1C

GSEA was executed to investigate the functions of CDKN1C. HALLMARK gene sets and KEGG pathways were considered as significantly enriched function annotations (*p* < 0.05, enrichment score >2.0). Furthermore, Through Tumor Immune Estimation Resource (TIMER) was applied to explore the association between CDKN1C expression and six essential TIICs. In order to determine whether the TIM differs markedly in low/high CDKN1C expression group, we used CIBERSORT, an established computational resource, to explore gene expression profiles of TCGA samples above to determine the levels of 22 immune cell subtypes in different CDKN1C expression groups. Finally, the association of 22 TIICs subtypes was analysed by Pearson Test.

To profile the variation of CDKN1C in BC, cBioportal was used to analyse the BC samples in TCGA Pan‐cancer Atlas. The CDKN1C genetic alteration in Chinese BC patients was also analysed to make a comparison. Acquired from patients who were diagnosed as invasive BC at the GDPH, 589 BC samples underwent next‐generation sequencing. It was approved by the Ethics Committee of GDPH and informed consents were obtained from all patients. Besides mRNA levels, differential protein expression between normal and BC tissues was validated by immunohistochemistry (IHC) staining obtained from the human protein atlas (HPA) database. HPA database retrieves transcriptomics data from TCGA and generates proteomics data. Therefore, using IHC analysis based on tissue microarrays, the transcriptomes of different human cancer types were visualized.

### 2.4 Construction and evaluation of CDKN1C‐based prognostic nomogram

To assist in clinical decision making, an applicable and quantitative model is required for predicting OS for BC patients. In terms of the multivariate analysis above, CDKN1C, age, TNM stage and tumour subtype were proved to be independent prognostic factors in BC survival. Therefore, we introduced a prognostic model integrating CDKN1C expression level and other clinicopathological factors. The area under the curve (AUC) of the receiver operating characteristic (ROC) curve for OS was formulated to assess the discrimination of the CDKN1C‐based model. As for its calibration ability, a calibration curve was drawn to verify. Finally, considering the potential for clinical utility, decision curve analysis (DCA) was used to assess the clinical practicability of the CDKN1C‐based nomogram.

### 2.5 Therapeutic responses estimation in BC patients

In the light of the optimal cut‐off value of CDKN1C‐based nomogram score, BC patients were divided into the high‐risk and low‐risk groups. High‐risk patients were characterized with higher scores and, therefore, worse predicted survival outcomes. Based on Genomics of Drug Sensitivity in Cancer, ‘Prophetic’ package was used to predict the therapeutic sensitivity. 6 common therapeutic agents for BC treatment were included. Their IC_50_ was estimated between two groups.

### 2.6 Statistical analysis

Descriptive analysis was conducted for clinicopathological features of included BC patients. Kaplan‐Meier curve and log‐rank test were adopted to plot and compare the survival curves. Univariate and multivariate analyses were used to verify the independent risk factors and construct a risk score formula and nomogram. Time‐dependent ROC curve analysis was exploited to evaluate the predictive accuracy of CDKN1C‐based nomogram. The calibration ability of the CDKN1C‐based nomogram was estimated using the calibration curve. Calibration plot was carried out to test the agreement between model‐predicted and actual outcome. The appropriate cut‐off values of CDKN1C expression level and CDKN1C‐based nomogram score were confirmed using X‐tile software, version 3.6.1 (Yale University, New Haven, CT, USA).^43^, ^44^ Statistical analyses were performed using R (Version 4.0.5) and a *p*‐value <0.05 was considered statistically significant.

## 3 RESULTS

### 3.1 Baseline characteristics

A total of 995 BC patients from TCGA database were included in our study. Median age of the patients selected was 58 years. The clinical and pathological characteristics are listed in Table 1, including T, N, TNM stage, ER, PR and HER2 status.

**TABLE 1:** Baseline characteristics of TCGA patients

Variables	Number (995)	%
Age (years)	58 (48, 67)	
T stage		
T1	268	26.9
T2	568	57.1
T3	122	12.3
T4	37	3.7
N stage		
N0	461	46.3
N1	340	34.2
N2	107	10.8
N3	70	7.0
Unknown	17	1.7
TNM Stage		
I	166	16.7
II	574	57.7
III	238	23.9
IV	17	1.7
ER status		
Negative	205	20.6
Positive	749	75.3
Unknown	41	4.1
PR status		
Negative	294	29.6
Positive	659	66.2
Unknown	42	4.2
HER2 status		
Negative	694	69.7
Positive	175	17.6
Unknown	126	12.7
Tumour subtype		
HR+/Her2‐	558	56.1
HR+/Her2+	139	14.0
HR‐/Her2+	36	3.6
TNBC	135	13.6
Unknown	127	12.8

Abbreviations: ER, estrogen receptor; HER2, human epithelial growth factor receptor 2; PR progesterone receptor; TNM, tumour‐node‐metastasis

### 3.2 Identification of CDKN1C signature in BC prognosis

**FIGURE 1:**
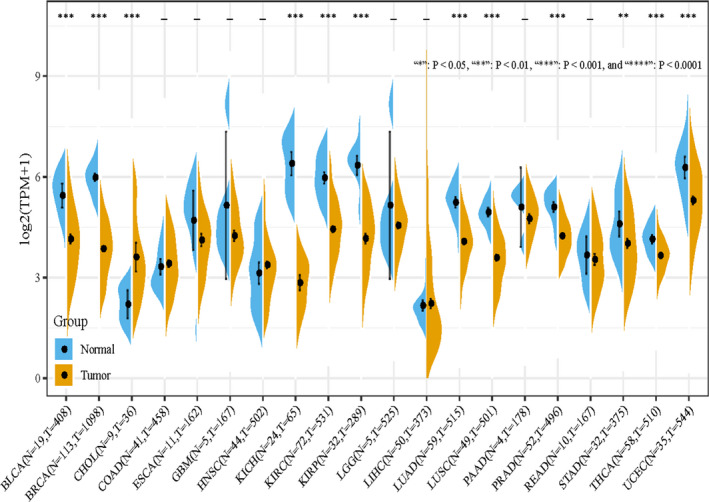
Differential expression levels of CDKN1C in multiple cancer types and normal tissues (‘∗’*p* < 0.05, ‘∗∗’*p* < 0.01, ‘∗∗∗’*p* < 0.001 and ‘∗∗∗∗’*p* < 0.0001)

**FIGURE 2:**
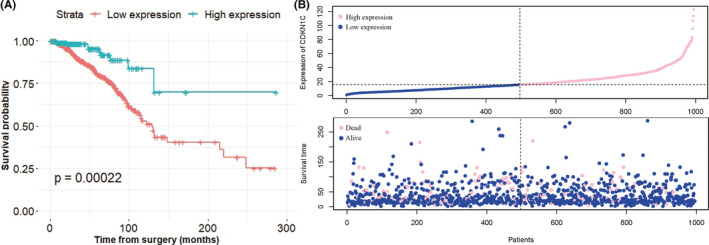
Kaplan–Meier curves of overall survival for breast cancer patients based on CDKN1C expression levels (A). CDKN1C expression and survival status distribution (B)

On the transcriptomic level, TCGA database analysis found that CDKN1C was significantly overexpressed in the normal tissue, compared with multiple tumours, such as BC, bladder urothelial carcinoma, kidney carcinoma and lung adenocarcinoma (Figure 1). Since the aberrant low expression of CDKN1C in BC, we further explored its prognostic value. In accordance with previous findings, Kaplan‐Meier survival analysis uncovered that BC patients with decreased levels of CDKN1C had a shortened OS (*p* = 0.00022, Figure 2A). The distribution of CDKN1C and survival status of the BC patients were shown in Figure 2B, indicating that its expression was positively correlated with the survival of BC patients. Then, CDKN1C expression and other clinicopathological factors were incorporated into univariate Cox proportional hazards regression analysis. Age, T, N and TNM stage, as well as subtypes displayed significant correlation with the prognosis of BC. Subsequently, above parameters were subjected to the multivariate Cox analyses. T and N stages were excluded since they were related with TNM stage and could result in spurious associations and unreliable results. As shown in Table 2, multivariate analyses identified CDKN1C (Hazard ratios (HR) =0.972, 95% confidence interval (CI) =0.956–0.988, *p* < 0.001) as an independent favourable prognostic factor for OS in BC patients. Moreover, age (HR =1.034, 95% CI =1.020–1.049, *p* < 0.001), stage (HR =1.523, 95% CI =0.859–2.701 for TNM stage II, *p* = 0.150; HR =3.680, 95% CI =2.031–6.666 for TNM stage III, *p* < 0.001; HR =6.756, 95% CI =2.831–16.122 for TNM stage IV, *p* < 0.001) and tumour subtype (HR =1.544, 95% CI =0.873–2.733 for HR+/HER2+, *p* = 0.136; HR =1.32, 95% CI =0.539–3.236 for HR‐/HER2+, *p* = 0.543; HR =1.962, 95% CI =1.157–3.327 for HR‐/HER2‐, *p* = 0.012) were verified as independent prognostic variables in BC patients.

**TABLE 2:** Univariate and multivariate Cox proportional hazards regression analyses in the TCGA patients

Variables	Univariate analysis		Multivariate analysis	
	Hazard ratios (95% CI)	*p*‐value	Hazard ratios (95% CI)	*p*‐value
Age	1.032 (1.018–1.047)	**<0.001**	1.034 (1.020–1.049)	**<0.001**
T stage				
T1	Referent	‐	‐	‐
T2	1.226 (0.801–1.876)	0.347	‐	‐
T3	1.250 (0.691–2.261)	0.46	‐	‐
T4	2.741 (1.438–5.226)	**0.002**	‐	‐
N stage				
N0	Referent	‐	‐	‐
N1	1.803 (1.188–2.736)	**0.006**	‐	‐
N2	2.610 (1.489–4.574)	**0.001**	‐	‐
N3	4.257 (2.254–8.038)	**<0.001**	‐	‐
Unknown	6.723 (3.244–13.931)	**<0.001**	‐	‐
TNM Stage				
I	Referent	‐	Referent	‐
II	1.459 (0.828–2.571)	0.191	1.523 (0.859–2.701)	0.15
III	3.013 (1.681–5.400)	**<0.001**	3.680 (2.029–6.658)	**<0.001**
IV	7.254 (3.166–16.619)	**<0.001**	6.716 (2.815–16.025)	**<0.001**
Tumour Subtype				
HR+/HER2‐	Referent		Referent	
HR+/HER2+	1.493 (0.848–2.628)	0.165	1.544 (0.872–2.731)	0.136
HR‐/HER2+	2.280 (0.974–5.336)	0.058	1.322 (0.539–3.239)	0.541
TNBC	1.561 (0.927–2.626)	0.094	1.960 (1.156–3.321)	**0.012**
Unknown	1.640 (1.041–2.585)	**0.033**	1.225 (0.772–1.945)	0.389
CDKN1C	0.971 (0.956–0.988)	**0.001**	0.972 (0.956–0.988)	**0.001**

Note

Bold values indicate the variable with statistically significance.

Abbreviations: HER2, human epithelial growth factor receptor 2; HR, hormone receptor; TNM, tumour‐node‐metastasis.

### 3.3 GSEA and genetic alteration analysis of CDKN1C

**FIGURE 3:**
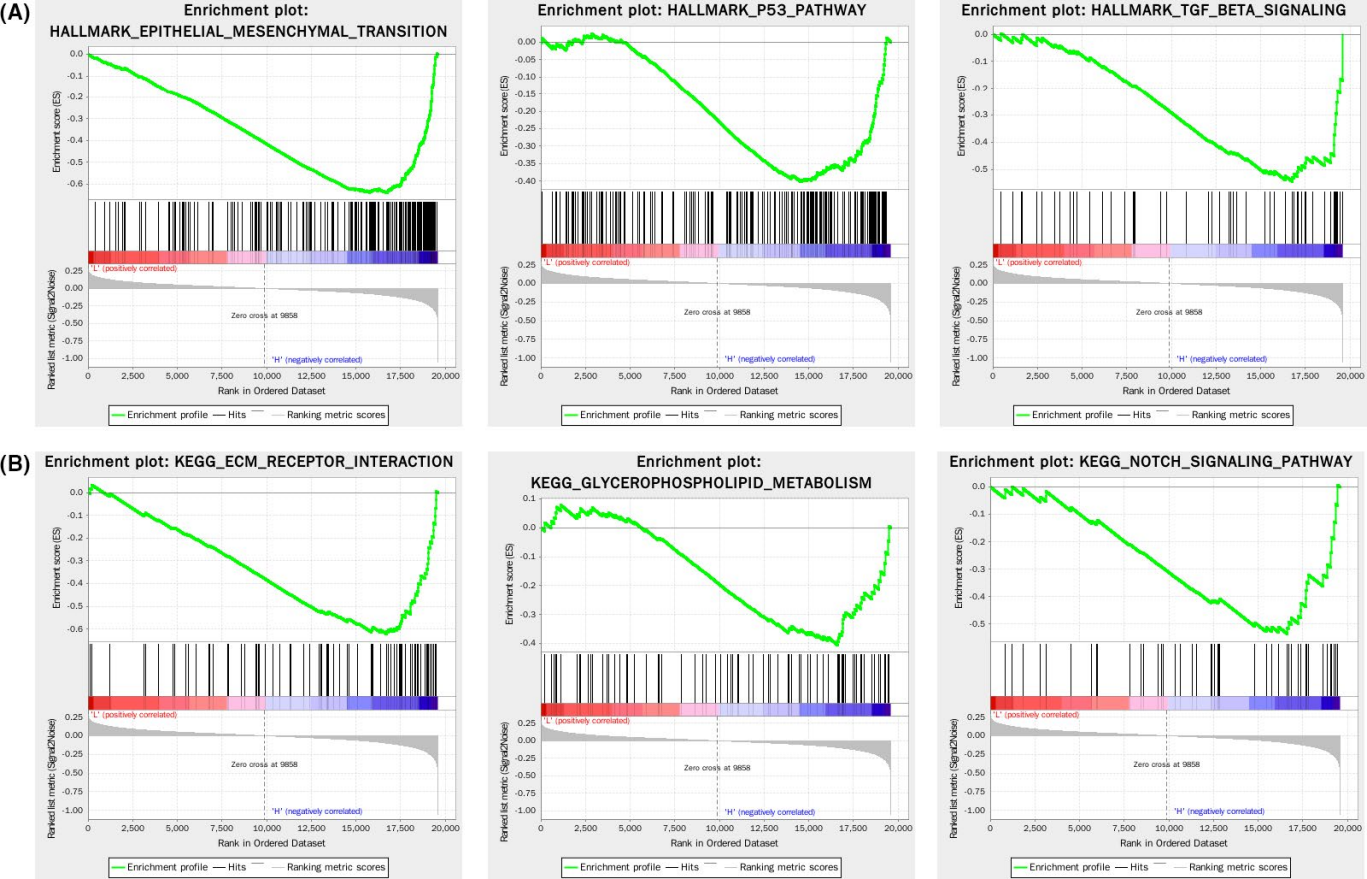
GSEA analyses of epithelial‐mesenchymal transition, p53 pathway and TGFβ signalling HALLMARK gene sets (A), KEGG pathways of ECM receptor interaction, glycerophospholipid metabolism and Notch signalling pathway (B) in breast cancer from TCGA

After exploring the correlation between CDKN1C expression levels and prognosis, GSEA was performed to clarify the biologic role of CDKN1C in BC progression (Figure 3). Hallmark gene sets exhibiting a strong negative correlation with CDKN1C were epithelial‐mesenchymal transition, p53 pathway and TGFβ signalling. Analogously, KEGG pathway analysis revealed significant enrichment of ECM receptor interaction, glycerophospholipid metabolism and Notch signalling pathway in the CDKN1C low‐expression group. These results suggested that the alteration of CDKN1C impacted BC tumorigenesis and development through proliferation, differentiation, migration or apoptosis.

**FIGURE 4:**
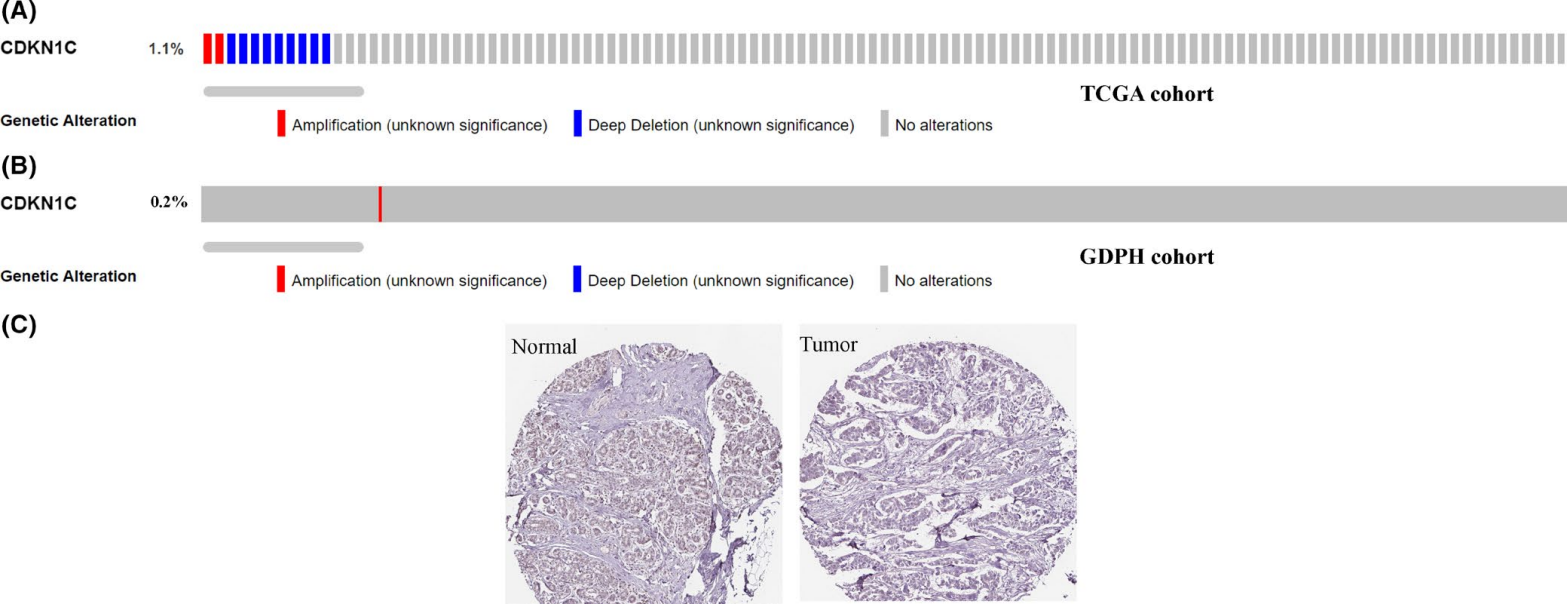
Genomic alteration profile of CDKN1C in breast cancer patients from TCGA database (A), GDPH patient cohort (B). Immunohistochemical validation of CDKN1C via HPA database (C)

After performing the functional analysis of CDKN1C, genetic alterations of CDKN1C in BC patients were followed. In patients obtained from TCGA portal, approximately 1.1% of the BC samples had mutations in CDKN1C (Figure 4A), of which 2 out of TCGA patients had amplification and 9 had deep deletion. As shown in Figure 4B, an amplification frequency of 0.2% was detected amongst the 589 BC samples in the Department of Breast Cancer, Guangdong Provincial People's Hospital (GDPH) cohort. These results unveiled that CDKN1C is rarely seen in BC. At the translational level, BC was validated to have a lower level of CDKN1C compared with normal breast tissues based on the HPA database (Figure 4C).

### 3.4 Relationship between CDKN1C expression and TIICs

**FIGURE 5:**
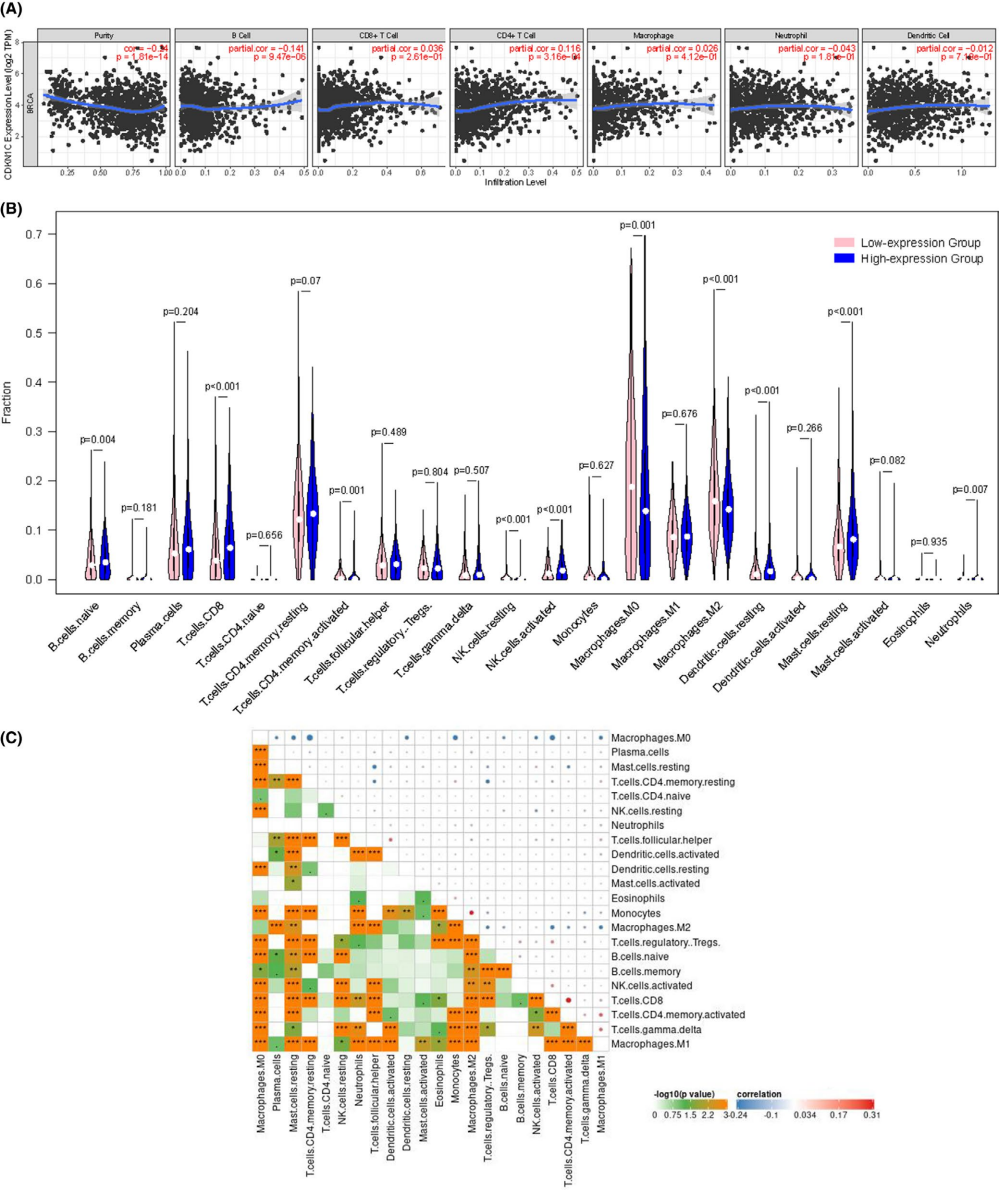
Correlation between CDKN1C expression and 6 TIICs (A). Differential proportions of 22 immune cell subtypes in low and high CDKN1C expression groups (B). Heatmap of 22 TIICs in breast cancer (C)

TIMER (Figure 5A), we observed that B cells were negatively correlated with CDKN1C (*p* = 9.47 × 10^−6^). Simultaneously, a positive correlation existed between its expression and CD4+ T cells (*p* = 3.16 × 10^−4^). It is noteworthy that with augmentation in CDKN1C expression, the tumour purity was significantly lower, indicating higher levels of TIICs. As shown in Figure 5B, incremental differences of activated CD4 memory T cells, M0 macrophages, M2 macrophages and resting NK cells were assessed in the low‐expression group. Inversely, the levels of naïve B cells, CD8 T cells, activated NK cells, resting dendritic cells, resting mast cells and neutrophils decreased when CDKN1C was downregulated. Figure 5C presented the correlation between 22 subtypes of TIIC in BC. A significant positive correlation existed between M2 macrophages and monocytes, CD8 T cells and activated CD4 memory T cells. M0 macrophages were found to be negatively associated with resting CD4 memory cells, CD8 T cells and monocytes.

### 3.5 Development and assessment of CDKN1C‐based prognostic model

**FIGURE 6:**
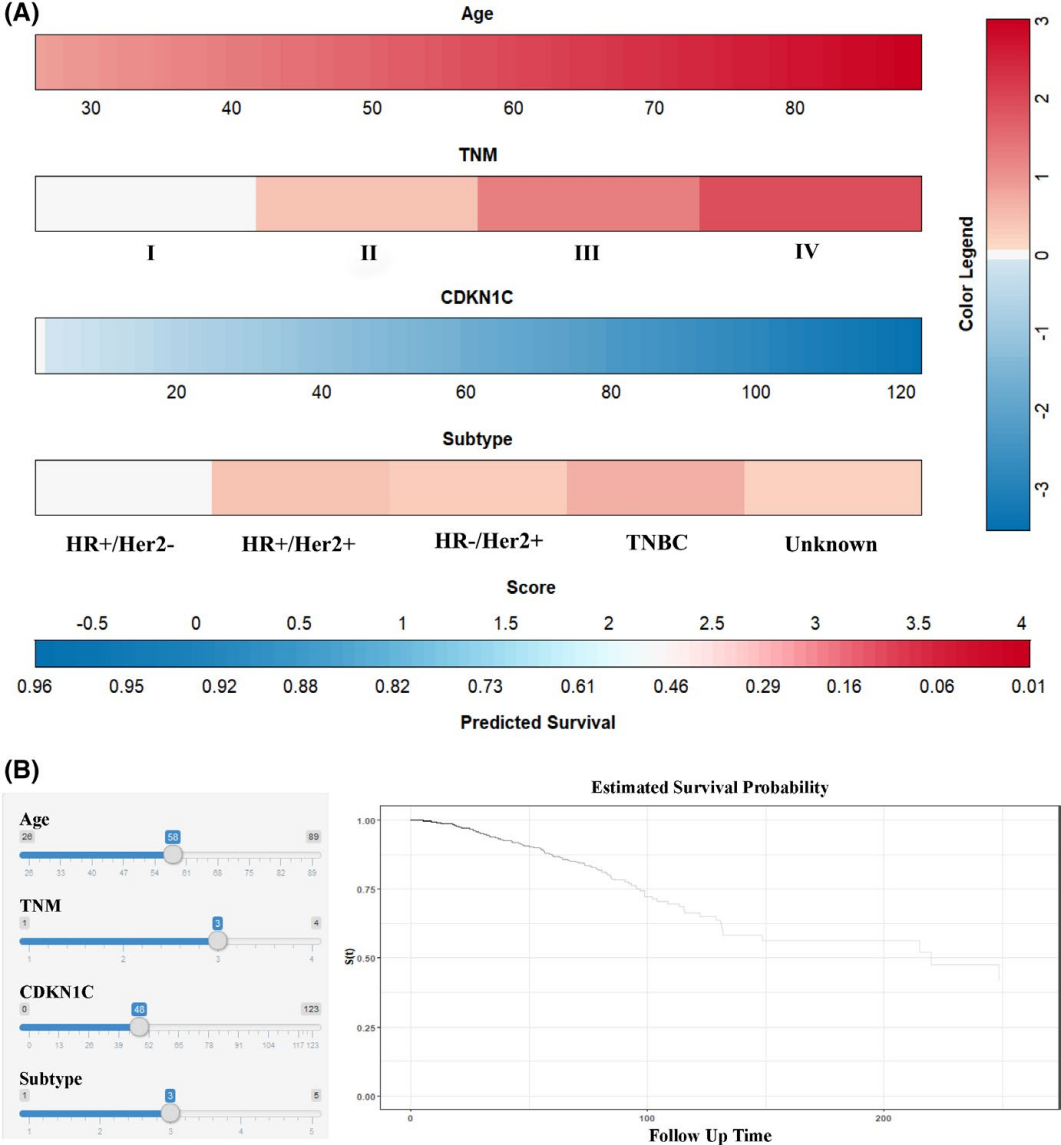
CDKN1C‐based prognostic model to predict 5‐year overall survival in breast cancer patients

**FIGURE 7:**
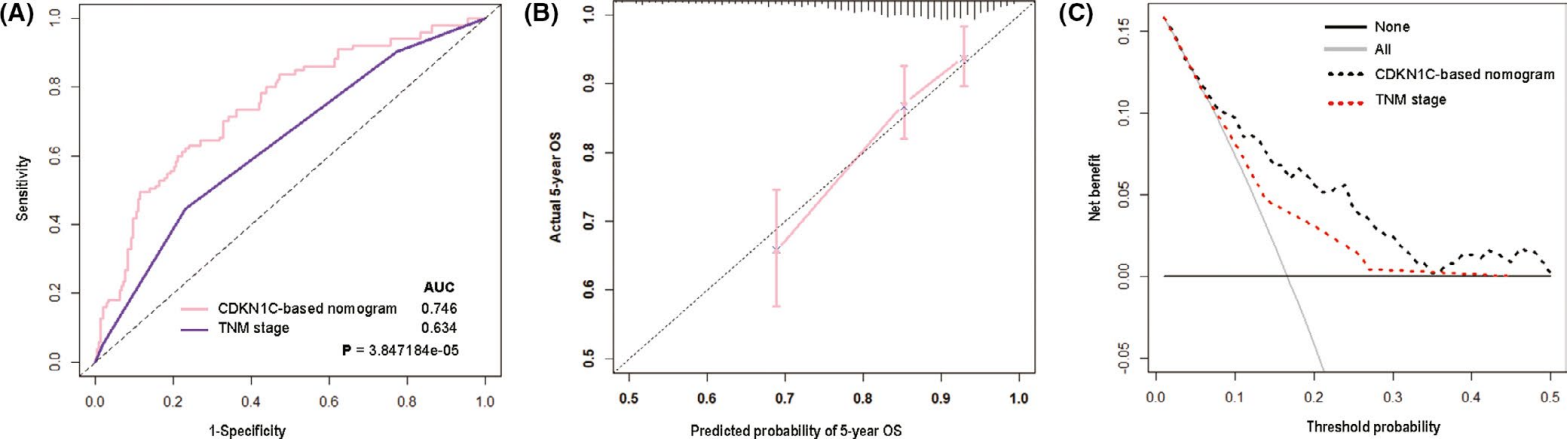
Comparison of the prognostic accuracy at 5‐year using time‐dependent ROC curves between the models with TNM stage (A). Calibration curves of the model in the TCGA cohort (B). Comparison of clinical utility using decision curve between the models with TNM stage (B)

Now, that CDKN1C level is related with survival outcomes probably due to the biological process and immune microenvironment, it may assist in prognosis prediction. Consequently, we established a nomogram incorporating the CDKN1C expression, age, TNM staging and tumour subtype aiming to predict the OS in BC patients (Figure 6). After calculating the nomogram score for each variable on the point scale, the final total score was gained to predict the 5‐year survival probability individually. Time‐dependent ROC analysis was utilized to evaluate the predictive accuracy of CDKN1C‐based prognostic model (Figure 7A). The AUC value of the nomogram for 5‐year survival rates prediction were 0.746 (95% CI: 0.677–0.816), in comparison with 0.634 (95% CI: 0.568–0.701) for the TNM staging system alone. A significantly better discrimination performance was exhibited (*p* < 0.001). Calibration plot displayed a strong conformity between the likelihoods generated by the nomogram and the actual results of 5‐year OS, suggesting high calibration ability (Figure 7B). DCA, as shown in Figure 7C revealed that the CDKN1C‐based model added more net benefit than did the traditional TNM stage, thus showing superior clinical practicability.

### 3.6 The role of nomogram in prediction of therapy sensitivity in BC patients

**FIGURE 8:**
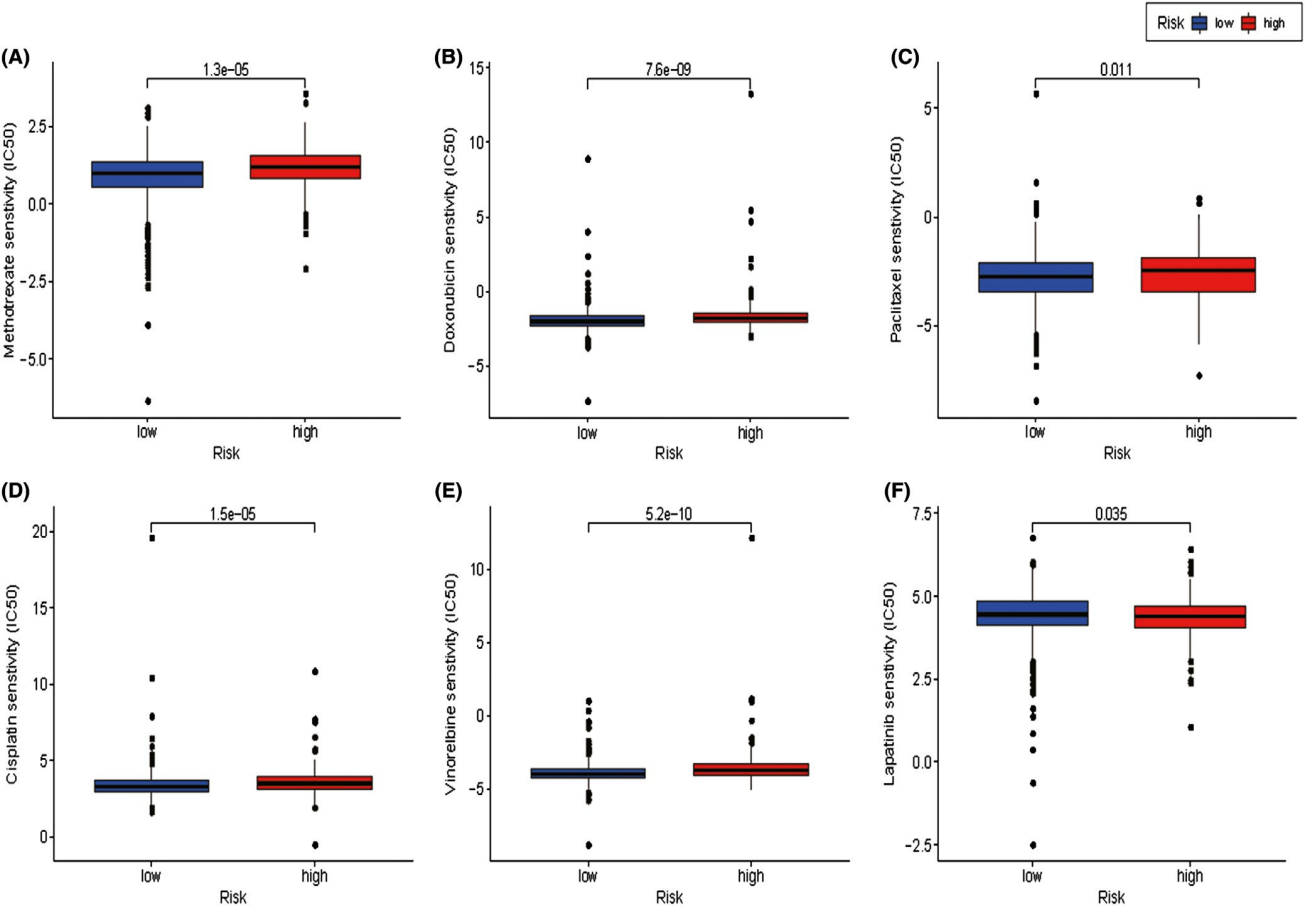
The box plots of the estimated IC_50_ for methotrexate (A), doxorubicin (B), paclitaxel (C), cisplatin (D), vinorelbine (E) and lapatinib (F) between low‐ and high‐risk groups

Finally, therapeutic response prediction was performed to compare BC patients in the low‐risk and high‐risk groups, with low and high nomogram scores respectively. In Figure 8, the estimated IC_50_ of methotrexate, doxorubicin, paclitaxel, cisplatin, vinorelbine were significantly reduced in the low‐risk group, which indicated better response to these therapeutic agents. Oppositely, lapatinib sensitivity was moderately better, when the nomogram scores were higher indicating worse prognosis.

## 4 DISCUSSION

Identification of a novel predictive signature is urgent for survival outcomes and therapeutic selection in BC survivors. CDKN1C, known as a BC suppressor, is transcriptionally and translationally expressed in the myoepithelial layer in BC.^45^ Kobatake et al. have uncovered the antioncogenic role of CDKN1C in BC.^28^ Hence, its prognostic role in BC has aroused interest of the subsequent researchers. For example, Yang and colleagues discovered that CDKN1C downregulation is correlated with poor survival in BC,^32^ which was limited by a small amount of samples and insufficient follow‐up data. Another study based on the TCGA and Oncomine data sets confirmed CDKN1C’s role in tumorigenesis and prognosis prediction.^36^ Our research confirmed the results of previous study, suggesting diminished expression of CDKN1C indicated unfavourable clinical outcomes in BC. We further explore the implication of CDKN1C in biological function and tumour immune infiltration. BC is known to be infiltrated by extensive immune cells that execute different roles to influence the cancer progression. Plentiful studies have verified the association between robust lymphocytic infiltration and favourable prognosis in cancers.^9^, ^46^ However, no evidence has put forward the potential effects of differential TIICs in BC genesis, according to the CDKN1C expression levels. CD8 cytotoxic lymphocyte is one representative that displays an antitumor role via cell‐mediated immune response and confers better clinical outcomes.^47^, ^48^ Its positive association with CDKN1C expression level, agreed with the favourable prognostic effect in the high‐expression group.

Although CDKN1C was identified as prognostic marker in BC previously, this signature has not been utilized to improve BC prognostic and therapeutic prediction. No evidence has revealed its predictive value for survival outcome. For the first time, we develop a novel predictive tool to unravel the prognostic significance of differential CDKN1C expression in BC. Besides traditional TNM staging system, molecular subtypes and age, we integrated CDKN1C levels aimed to achieve the sufficient survival assessment for this heterogeneous cancer. Satisfactorily, the nomogram yielded favourable discrimination and calibration in BC prognosis prediction and conferred superior clinical benefit than TNM stage alone. Furthermore, the nomogram had better ability to predict therapeutic responses than previous tools, thus providing personalized treatment regimen clinically. Despite the survival outcome has been improved in recent years, BC recurrence frequently occurs due to drug resistance. Thus, effective biomarkers to assess therapeutic responses for BC patients remain imperative in clinical practice. Although previous studies tried to find out some predictive markers for chemotherapy response,^7^, ^49^ the scantiness of drugs variety limited its guidance for drug selection. It was not accurate enough to untangle the heterogeneity of BC treatment response by assessment of intrinsic clinicopathological features or genes expression solely.^23^, ^50^, ^51^ Taking together, our nomogram was utilized to distinguish the low‐risk and high‐risk patients with different drugs sensitivities. We found that BC patients with high nomogram scores in high‐risk groups manifested stronger sensitivity to lapatinib. On the contrary, low‐risk patients were more sensitive to methotrexate, doxorubicin, paclitaxel, cisplatin, vinorelbine. In the late 1990s, classic CMF (cyclophosphamide, methotrexate and 5‐fluorouracil) was widely used in BC treatment^52^ and subsequently moved to anthracycline‐based regimens, represented by four cycles of doxorubicin and cyclophosphamide.^53^, ^54^ With the advent of new antimicrotubule agents, paclitaxel has later become the standard‐of‐care drug in early BC and significantly improved survival outcomes.^52^, ^55^ Compared with anthracyclines and taxanes, superiority of cisplatin and vinorelbine was reported in some metastatic BC.^56^, ^57^, ^58^ Nevertheless, adverse effects of chemotherapy can overshadow their acknowledged efficacy.^59^ Therefore, we supposed this CDKN1C‐based nomogram to select a subset of patients most likely to benefit from lapatinib or the addition of these therapeutic agents. To sum up, the nomogram is able to improve the accuracy of prognosis prediction for BC patients and identifying the potential cohorts, thus providing appropriate systemic therapy and follow‐up strategies.

In addition, there are some limitations in the present research. Firstly, our analysis was mainly based on online databases. The in‐vivo and in‐vitro experiments are required to explore the mechanism of CDKN1C on BC progression, signalling pathways and immune regulatory function in the future. Secondly, the CDKN1C‐based nomogram should be verified by the prospective, large‐scale cohorts before clinical application. Moreover, larger clinical trials to validate the role of CDKN1C‐based nomogram in antitumor drugs selection are needed.

In summary, a novel CDKN1C‐based nomogram was developed to estimate the survival outcome of BC patients, which reflected good predictive accuracy and outperformed the TNM staging alone. At the same time, we can find out the patients who may maximally benefit from specific antitumor agents, thus reducing the burden of overtreatment. Our study provided new insights into the role of CDKN1C, and facilitate prognosis and therapeutic responses prediction.

## CONFLICT OF INTEREST

The authors declare that they have no competing interests.

## AUTHOR CONTRIBUTIONS

**Jianguo Lai:** Conceptualization (lead); Project administration (lead); Writing‐original draft (lead); Writing‐review & editing (lead). **Xiaoyi Lin:** Formal analysis (lead); Investigation (lead); Supervision (lead). **Fangrong Cao:** Data curation (lead); Investigation (lead); Resources (lead); Software (lead); Visualization (lead). **Hsiaopei Mok:** Data curation (lead); Resources (lead); Software (lead). **Bo Chen:** Conceptualization (lead); Funding acquisition (lead); Project administration (lead); Writing‐original draft (lead); Writing‐review & editing (lead). **Ning Liao:** Conceptualization (lead); Funding acquisition (lead); Project administration (lead); Writing‐original draft (lead); Writing‐review & editing (lead).

## Data Availability

The data sets used and analysed during the current study are available from the corresponding author on reasonable request.
